# Pain management program outcomes in veterans with chronic pain and comparison with nonveterans

**DOI:** 10.1080/24740527.2020.1768836

**Published:** 2020-08-04

**Authors:** Jane Jomy, Eleni G. Hapidou

**Affiliations:** a Michael G. DeGroote Pain Clinic, McMaster University Medical Centre, Hamilton, Ontario, Canada; b Department of Psychiatry and Behavioural Neurosciences, McMaster University, Hamilton, Ontario, Canada

**Keywords:** chronic pain, veterans, pain management, psychological assessment, pain assessment, pain treatment

## Abstract

**Background**: In Canada, 41% of veterans experience chronic pain compared to the general population (20%). Many veterans with chronic pain also have comorbid disorders such as depression and posttraumatic stress disorder (PTSD), causing increased pain interference and disability.

**Aim**: This study aims to investigate the effectiveness of a 4-week interdisciplinary pain management program at the Michael G. DeGroote Pain Clinic in Hamilton, Ontario, Canada, and to explore differences in pain experience and treatment outcomes between veterans and nonveterans in the program.

**Methods**: Data were obtained from psychometric measures completed by 68 veterans and 68 nonveterans enrolled in the pain management program. By matching groups for age and gender, scores were compared between veterans and nonveterans. Outcomes investigated include catastrophizing, pain traumatization, stages of change, acceptance of pain, and program satisfaction. Multivariate analysis of variance (MANOVA) was conducted to examine session (admission–discharge) and group (veteran–nonveteran) differences, and independent *t* tests were used to examine differences in satisfaction measures.

**Results**: Results showed that the program was effective for all participants, with significant differences between admission and discharge on several measures. However, veterans experienced significantly greater improvements in pain catastrophizing, kinesiophobia, pain traumatization, pain acceptance, stages of change, and pain coping, compared to nonveterans (*P < *0.05). Though no significant differences in program satisfaction were found between groups, case managers evaluated veterans as having achieved greater benefits from the program.

**Conclusion**: This study presents evidence supporting the effectiveness of an interdisciplinary pain management program in addressing pain-related variables in veterans and nonveterans and provides insight into how pain management is experienced differently by veterans.

## RÉSUMÉ

**Contexte**: Au Canada, 41 % des anciens combattants souffrent de douleur chronique, comparativement à 20 % au sein de la population en général. De nombreux anciens combattants souffrant de douleur chronique sont également atteints de troubles comorbides tels que la dépression et le syndrome de stress post-traumatique, qui entraînent une augmentation de l'interférence de la douleur et de l'incapacité.

**Objectif**: Cette étude porte sur l'efficacité d'un programme de prise en charge interdisciplinaire de la douleur d'une durée de quatre semaines à la Clinique de la douleur Michael G. DeGroote à Hamilton, Ontario, Canada, ainsi que sur les différences dans l'expérience de la douleur et les résultats des traitements entre les anciens combattants et d'autres patients qui n'étaient pas des anciens combattants inscrits au programme.

**Méthodes**: Les données ont été obtenues à partir de mesures psychométriques recueillies auprés de 68 anciens combattants et de 68 patients qui n'étaient pas des anciens combattants, tous inscrits au programme de prise en charge de la douleur. En faisant correspondre les groupes selon l'âge et le sexe, les scores obtenus par les anciens combattants ont été comparés à ceux des autres patients qui n'étaient pas des anciens combattants. Les résultats étudiés comprenaient la catastrophisation, le traumatisme lié à la douleur, les étapes du changement, l'acceptation de la douleur et la satisfaction à l'égard du programme. Une analyse multivariée de la variance (MANOVA) a été effectuée pour examiner les différences entre les sessions (admission-sortie) et entre les groupes (anciens combattants et patients qui n'étaient pas des anciens combattants), et des tests t indépendants ont été utilisés pour examiner les diff'rences dans les mesures de satisfaction.

**Résultats**: Les résultats ont montré que le programme était efficace pour tous les participants, et que des différences entre l'admission et la sortie étaient observées pour plusieurs mesures. Cependant, les anciens combattants ont connu des améliorations nettement plus importantes en ce qui concerne la catastrophisation de la douleur, la kinésiophobie, le traumatisme lié à la douleur, l'acceptation de la douleur, les stades de changement et la prise en charge de la douleur, comparativement aux patients qui n'étaient pas des anciens combattants (P < 0,05). Bien qu'aucune différence significative dans la satisfaction à l'égard du programme n'ait été constatée entre les groupes, les chargés de cas ont évalué que les anciens combattants avaient tiré plus d'avantages du programme.

**Conclusions**: Cette étude présente des preuves de l'efficacité d'un programme interdisciplinaire de lutte contre la douleur en abordant les variables liées à la douleur chez les anciens combattants et chez des patients qui n'étaient pas des anciens combattants. Elle donne aussi un aperçu de la maniére dont la prise en charge de la douleur est vécue différemment par les anciens combattants.

## Introduction

Chronic pain is one of the most common reasons adults seek medical treatment, producing a significant economic and social burden. Living with chronic pain results in interference with physical functioning, daily activities, mental health, and social and family functioning.^1^ Studies have demonstrated that chronic pain is more prevalent in certain populations, such as low socioeconomic groups, racial and ethnic minorities, veterans, and the elderly.^2^ In Canada, one in five adults have reported experiencing chronic pain, and as the aging population is growing, so is the magnitude of this major health problem.^3^

Among U.S. war veterans, up to 50% of men and 78% of women reported chronic pain.^4^ In Canadian veterans, survey results demonstrate that 41% experienced constant chronic pain and 25% reported pain interference, highlighting the importance of studying the unique experience of chronic pain in this population.^5^ Not only do veterans have a higher prevalence of chronic pain but they also report higher rates of severe pain.^6^

Investigating comorbidity with posttraumatic stress disorder (PTSD) is important in veterans because 50.1% of veterans diagnosed with PTSD also report chronic pain.^7^ Research suggests that when participating in pain management programs, patients with comorbid PTSD and chronic pain experience a reduction in PTSD severity, whereas pain-related symptoms (i.e., pain intensity and interference) do not improve.^8^

Many models attempt to explain the relationship between chronic pain and PTSD. One of these models was proposed by Sharp and Harvey, the mutual maintenance model, which posits that the behavioral, affective, and cognitive features of each condition can exacerbate the other.^9^ This model identifies seven processes for the joint preservation of chronic pain and PTSD, including attentional biases, anxiety sensitivity, pain-related triggers, avoidance behaviors, fatigue, general anxiety, and cognitive demands.^9^ Further, the shared vulnerability model, proposed by Asmundson et al., expands on the mutual maintenance model by suggesting that the features of each condition become vulnerability factors for developing the conditions in addition to exacerbating each other.^10,11^

To better understand how veterans experience pain differently from other individuals with chronic pain, scores from psychometric tests such as the Pain Catastrophizing Scale (PCS) and the Chronic Pain Coping Inventory (CPCI) can be used by pain management programs for assessment and evaluation of pain-related outcomes. A previous study conducted by Jiwani and Hapidou on a multidisciplinary chronic pain program provided evidence of effectiveness as scores on pain catastrophizing, task persistence, and seeking social support coping strategies improved among veterans.^12^

Investigating program outcomes is beneficial for both veterans and nonveterans because chronic pain greatly reduces an individual’s quality of life. The current study was conducted with the following hypothesis: Both veterans and nonveterans are expected to demonstrate an improvement in scores at discharge, illustrating the effectiveness of the pain management program.

## Materials and Methods

### Participants

This retrospective study uses archival data from patients (*n = *136) who have completed the 4-week Michael G. DeGroote Pain Clinic–Intensive Chronic Pain Management Program (MGD 4-week program) in Hamilton, Ontario, Canada, from the opening of the clinic in 2015 until August 2019. During this time, all patients in the study completed the same psychometric packages. The MGD 4-week program is an interdisciplinary pain management program based on the biopsychosocial model of assessment, prevention, and treatment of chronic pain. Interventions included fitness, psychoeducation, group therapy, pharmacy assessment, relaxation and mindfulness sessions, yoga, tai chi, hydrotherapy, and social work, among other sessions. Individual adjustments were made occasionally to the program schedule because some chose not to attend hydrotherapy, for example, and instead added relaxation or individual sessions as needed. Each patient’s care team consisted of a physician, psychologist, psychometrist, social worker, physiotherapist, occupational therapist, pharmacist, dietician, and support staff. At initial assessment, all participants consented to the use of their information for research and program quality improvement purposes by signing an informed consent form. The study was approved by the Hamilton Integrated Research Ethics Board (Project Number 7421-C).

All veterans (*n = *68) who completed the MGD 4-week program with complete psychometric measures were included in the study and were matched to nonveterans (*n = *68) based on gender and age only.

### Measures

All patients completed a psychometric package at program admission and discharge to provide the health care team with an improved understanding of the patient’s experience with pain.

#### Pain Intensity Scale

The Pain Intensity Scale measures pain intensity in the past 2 weeks on a scale of 0 (*no pain*) to 10 (*unbearable pain*). The Pain Intensity Scale is a valid and reliable measure of pain intensity and has strong associations with the Pain Disability Index (PDI) and other pain measures.^13–16^

#### Center for Epidemiological Studies Depressed Mood Scale

The Center for Epidemiological Studies Depressed Mood Scale (CES-D) was used to assess symptoms of depressed mood experienced in the past week.^17^ The CES-D is made up of 20 items, with response options ranging from 0 (*rarely or none of the time*) to 3 (*most or all of the time*). The CES-D has been found to be a valid measure of depressive symptoms in the general and chronic pain populations. Moreover, the CES-D has demonstrated superior sensitivity in identifying differences in depression severity when compared to other depression scales (i.e., Beck Depression Inventory).^18^

#### Pain Catastrophizing Scale

The PCS was used to assess catastrophic thinking, which is defined as an exaggerated negative orientation toward the threat of actual or anticipated pain.^19^ There are 14 items with response options of 0 (*not at all*) to 4 (*all of the time*) that describe various perceptions and feelings that individuals may have regarding their pain and pertain to one of three subscales: Rumination, Magnification, and Helplessness. The PCS has demonstrated convergent validity with self-reported anxiety measures and strong test–retest reliability.^20^

#### Clinical Anxiety Scale

The Clinical Anxiety Scale (CAS) measures participants’ current level of anxiety with 25 items. Response options range from 1 (*rarely or none of the time*) to 5 (*most or all of the time*). The CAS is a very reliable and valid measure with high internal consistency (*α = *0.94) and good discriminant validity (*r* = 0.77).^21^

#### Patient Questionnaire of the Primary Care Evaluation of Mental Disorders

The Patient Questionnaire of the Primary Care Evaluation of Mental Disorders patient questionnaire (PQ) assesses physical and emotional symptoms experienced in the past month with 25 true or false questions and a self-rating of overall health as *excellent, very good, good, fair*, or *poor*. Research has demonstrated that the PQ is a useful tool in screening mental disorders, demonstrating good to excellent sensitivity across all diagnoses: mood (69%), anxiety (94%), alcohol (81%), and eating disorders (86%).^22^

#### Pain Disability Index

The PDI measures the extent to which chronic pain interferes with family and home responsibilities, recreation, social activity, occupation, sexual behavior, self-care, and life support activity. Response options range from 0 (indicating no disability at all) to 10 (total disruption by pain).^23^ The PDI is a reliable measure, demonstrating high internal consistency and high reported test–retest reliability.^14^ Additionally, patients with higher PDI scores had significantly more pain characteristics such as psychological distress than patients with low PDI scores.^24^

#### Tampa Scale of Kinesiophobia

The Tampa Scale of Kinesiophobia (TSK) is the most widely used self-report measure of kinesiophobia in patients with chronic pain.^25^ A briefer and simpler version was created in 2005 by excluding six items with inadequate measurement properties (TSK-11).^26^ Response options range from 1 (*strongly disagree*) to 4 (*strongly agree*). The TSK-11 has been validated for patients with chronic low back pain and chronic musculoskeletal pain in adults and seniors.^26–29^

#### Chronic Pain Acceptance Questionnaire

The Chronic Pain Acceptance Questionnaire (CPAQ) measures acceptance of chronic pain with 20 items organized into two subscales, Activity Engagement and Pain Willingness. Response options range from 0 (*never true*) to 6 (*always true*). The CPAQ has demonstrated very good internal consistency (α = 0.82 for Activity Engagement and α = 0.78 for Pain Willingness).^30,31^ Additionally, the CPAQ has demonstrated predictive validity for depression, pain-related anxiety, and psychosocial disability.^31^

#### Pain Stages of Change Questionnaire

The Pain Stages of Change Questionnaire (PSOCQ) measures readiness to adopt self-management to chronic pain with 30 items divided between four subscales: Pre-contemplation, Contemplation, Action, and Maintenance. Participants rate how strongly they agree or disagree with statements using a scale from 1 (*strongly disagree*) to 5 (*strongly agree*).^32^ The PSOCQ has demonstrated very good to excellent reliability for each subscale: Pre-contemplation (α = 0.77), Contemplation (α = 0.82), Action (α = 0.86), and Maintenance (α = 0.86) and excellent test–retest reliability (α = 0.74–0.88).^32^

#### Chronic Pain Coping Inventory

The CPCI was used to assess participants’ use of coping strategies, with 70 items organized into nine subscales: Guarding, Resting, Asking for Assistance, Exercise Stretch, Relaxation, Task Persistence, Coping Self-Statements, Pacing, and Seeking Social Support.^33^ Participants endorse the number of days during the past week (0–7) on which they utilized each method. Research demonstrates very high correlations, internal consistency, and test–retest stability and high validity, suggesting that the CPCI is a valuable tool in assessing coping strategies for pain.^34^

#### Sensitivity to Pain Traumatization Scale

The Sensitivity to Pain Traumatization Scale (SPTS) measures anxiety-related cognitive, emotional, and behavioral reactions to pain that resemble the features of a traumatic stress reaction. This measure has 12 items rated on a 5-point scale ranging from 0 (*not at all true*) to 4 (*entirely true*). Research has demonstrated that the SPTS has excellent reliability and concurrent validity and moderate convergent and discriminant validity.^35^

#### Minnesota Multiphasic Personality Inventory-2

The Minnesota Multiphasic Personality Inventory-2 (MMPI-2) is the most widely used personality inventory in the clinical setting.^36,37^ It measures personality characteristics and psychopathology of individuals through 567 empirically validated true–false statements.^36,38^ The MMPI-2 contains nine validity scales: Variable Response Inconsistency (VRIN), True Response Inconsistency (TRIN), Infrequency (F), Back F (Fb), Psychopathology (Fp), Symptom Validity (FBS), Lie (L), Correction (K), and Superlative Self-Presentation (S). Validity scales accommodate for over/underreporting by measuring inconsistencies in responses. Participants (*n* = 16) who scored ≥80 on the VRIN scale and/or ≥100 on the F scale were excluded from MMPI-2 analysis. There are also ten clinical scales: Hypochondriasis (Hs), Depression (D), Hysteria (Hy), Psychopathic Deviate (Pd), Masculinity/Femininity (Mf), Paranoia (Pa), Psychasthenia (Pt), Schizophrenia (Sc), Hypomania (Ma), and Social Introversion (Si). The MMPI-2 was administered once at admission only.

#### Pain Program Satisfaction Questionnaire

The Pain Program Satisfaction Questionnaire (PPSQ) has 11 questions rated on a 4-point Likert scale and two open-ended questions to assess patient satisfaction.^37,39^ The reliability and validity of the PPSQ were first examined in a sample of 44 patients who completed the 4-week intensive pain management program (α = 0.87).^39^

#### Self-Evaluation Scale and Patient Evaluation Scale

The Self-Evaluation Scale (SES) asks patients to rate their goal accomplishment on a 5-point Likert scale ranging from 1 (*poorly*) to 5 (*excellent*).^37^ The current study found that the internal consistency of the SES (Cronbach’s alpha) was α = 0.81. Additionally, the correlation coefficient between the PPSQ and the patient’s self-evaluation was 0.83 (<0.001). The patient’s case managers also completed the Patient Evaluation Scale, identical to the SES but answering the question “How has your patient accomplished their goals in the past four weeks?” The Patient Evaluation Scale also demonstrated high internal consistency (α = 0.89).

### Data Analysis

This study has a between-subjects and within-subjects design. The independent variables are group (veteran and nonveteran) and session (admission and discharge). The dependent variables are psychometric test scores at admission and discharge, MMPI-2 scores (admission only), and discharge-only questionnaire scores.

Independent *t* tests and chi-square tests were conducted using SPSS 26.0 to determine statistically significant (*P < *0.05) differences in demographic characteristics. Multivariate analysis of variance (MANOVA) was used to determine statistically significant (*P* < 0.05) differences in mean psychometric test scores. A main group effect was demonstrated when there was a difference between veterans and nonveterans at both admission and discharge on average. A main session effect was demonstrated when there was a difference between admission and discharge for all participants on average. When a combination of group and session differences are greater together than alone, a group by session interaction effect occurs. An example of an interaction effect was when a difference between admission and discharge was only found among veterans. Significant group by session interaction effects were further examined by post hoc multiple comparisons (Scheffe tests) as summarized in Table A1 (see Appendix A). MMPI-2 subscale scores and discharge questionnaire scores were compared using *t* tests.

## Results

### Demographics

Table 1 summarizes the demographics of all participants. Though veterans and nonveterans are similar on many demographic characteristics, there are a few key differences. All veterans were referred to the program by Veterans Affairs Canada; all nonveterans were referred from other sources such as motor vehicle accident insurance companies and the Workplace Safety and Insurance Board. Veterans had a significantly greater number of injuries related to work and visits to their family physician compared to nonveterans. In addition, a greater number of veterans were unemployed (and pensioned off) at program admission than nonveterans. Another key difference found is pain chronicity, the number of years since the pain problem began, as reported by patients at admission. Pain chronicity was found to be significantly correlated with group membership (*P < *0.001) because veterans had pain for an average of 16.8 years, whereas nonveterans had pain for an average of 5.0 years (see Appendix B).

**Table 1.** Patient demographics

	Veterans	Nonveterans
Mean age (SD)	48.5 (9.5)	48.3 (12.6)
Gender	Men (*n* = 56) Women (*n* = 12)	Men (*n* = 56) Women (*n* = 12)
Referral source*	VAC (*n* = 68) Insurance (*n* = 0) WSIB (*n* = 0) OHIP (*n* = 0) Unknown (*n* = 0)	VAC (*n* = 0) Insurance (*n* = 43) WSIB (*n* = 9) OHIP (*n* = 9) Unknown (*n* = 3)
Mean number of years since last employed (SD)	3.7 (3.8)	4.4 (5.9)
Mean number of years since pain problem began (SD)**	16.8 (12.1)	5.0 (5.0)
Mean number of visits to the emergency room (SD)	3.9 (3.9)	2.8 (3.4)
Children	None (*n* = 13) 1–2 (*n* = 33) 3+ (*n* = 22)	None (*n* = 11) 1–2 (*n* = 39) 3+ (*n* = 18)
Marital status	Single (*n* = 8) Married/common-law (*n* = 51) Separated/divorced (*n* = 6)	Single (*n* = 15) Married/common-law (*n* = 50) Separated/divorced (*n* = 11)
Employment status at start of the program*	Employed (*n* = 7) Unemployed (*n* = 61)	Employed (*n* = 26) Unemployed (*n* = 38)
Number of injuries related to work^a^*	0 injuries (*n* = 1) 1 injury (*n* = 12) 2 injuries (*n* = 9) 3+ injuries (*n* = 45)	0 injuries (*n* = 25) 1 injury (*n* = 10) 2 injuries (*n* = 9) 3+ injuries (*n* = 7)
Number of injuries for other reasons (e.g., car accident)^a^*	0 injuries (*n* = 21) 1 injury (*n* = 7) 2 injuries (*n* = 6) 3+ injuries (*n* = 6)	0 injuries (*n* = 7) 1 injury (*n* = 16) 2 injuries (*n* = 6) 3+ injuries (*n* = 14)
Number of visits to family physician^a^*	1–4 visits (*n* = 4) 5–9 visits (*n* = 6) 10–19 visits (*n* = 11) 20+ visits (*n* = 40)	1–4 visits (*n* = 17) 5–9 visits (*n* = 16) 10–19 visits (*n* = 12) 20+ visits (*n* = 9)
Number of visits to other specialists^a^	0–4 visits (*n* = 34) 5–9 visits (*n* = 20) 10+ visits (*n* = 12)	0–4 visits (*n* = 20) 5–9 visits (*n* = 18) 10+ visits (*n* = 17)
PTSD (diagnosis, features, or query)*	Yes (*n* = 43) No (*n* = 25)	Yes (*n* = 25) No (*n* = 34)

^a^Since pain problem began.

*Indicates a significant chi-square test result (*P < *0.05).

**Indicates a significant *t* test result (*P < *0.05).

VAC = Veterans Affairs Canada; WSIB = Workplace Safety and Insurance Board; OHIP = Ontario Health Insurance Plan; PTSD = posttraumatic stress disorder.

### Program Evaluation

Table 2 compares average scores on psychometric measures completed at admission and discharge between veterans and nonveterans. Multivariate tests revealed the following Wilks’ lambda values for group (veteran–nonveteran), 0.6, *F*(23, 51) = 1.7, *P < *0.001, session (admission–discharge), 0.1, *F*(23, 51) = 20.3, *P < *0.001, and group by session interactions, 0.5, *F*(23, 51) = 1.9, *P < *0.001.

**Table 2.** Comparison of scores between veterans and nonveterans at admission and discharge

Session	*n*^a^	Veteran average (SD)	Nonveteran average (SD)	Cohen’s *d*	*P*	*F*
Pain Intensity Scale						
Admission Discharge	39	5.5 (1.6) 5.1 (1.6)	5.7 (1.5) 5.5 (1.9)	0.2 0.3	Group = 0.31 Session = 0.10 Interaction = 0.5	
Center for Epidemiological Studies Depressed Mood Scale						
Admission Discharge	62	32.3 (10.6) 19.5 (10.7)	34.2 (11.1) 24.4 (12.5)	0.2 0.4	Group = 0.06 Session = 0.00 Interaction = 0.09	Session *F*(1, 61) = 4.27, *P* < 0.001
Pain Catastrophizing Scale						
Admission Discharge	62	30.5 (12.0) 16.1 (9.9)	33.2 (13.1) 25.6 (13.3)	0.2 0.8	Group = 0.02 Session = 0.00 Interaction = 0.01	Interaction *F*(1, 61) = 7.32, *P* = 0.01
Clinical Anxiety Scale						
Admission Discharge	63	42.4 (17.5) 29.9 (13.5)	42.0 (20.6) 37.6 (18.1)	0.02 0.5	Group = 0.21 Session = 0.00 Interaction = 0.00	Interaction *F*(1, 62) = 7.49, *P* < 0.001
Patient Questionnaire of the Primary Care Evaluation of Mental Disorders						
Admission Discharge	64	13.8 (3.7) 10.2 (4.1)	13.3 (3.8) 11.6 (4.6)	0.1 0.3	Group = 0.44 Session = 0.00 Interaction = 0.00	Interaction *F*(1, 63) = 1.73, *P* < 0.001
Pain Disability Index						
Admission Discharge	64	48.4 (8.1) 41.5 (10.1)	47.3 (9.9) 41.2 (12.1)	0.1 0.03	Group = 0.67 Session = 0.00 Interaction = 0.59	Session *F*(1, 63) = 4.70, *P* < 0.01
Tampa Scale of Kinesiophobia						
Admission Discharge	59	32.4 (5.9) 22.1 (6.0)	31.2 (7.0) 27.8 (7.8)	0.19 0.82	Group = 0.05 Session = 0.00 Interaction = 0.00	Interaction *F*(1, 58) = 18.31, *P* < 0.001
Chronic Pain Acceptance Questionnaire Activities of Engagement						
Admission Discharge	64	27.9 (10.5) 37.5 (8.6)	24.4 (12.2) 32.0 (11.1)	0.31 0.55	Group = 0.00 Session = 0.00 Interaction = 0.32	Group *F*(1, 63) = 19.64, *P* < 0.001 Session *F*(1, 63) = 8.61, *P* < 0.001
Chronic Pain Acceptance Questionnaire Pain Willingness						
Admission Discharge	64	18.1 (7.6) 22.3 (7.63)	16.0 (8.2) 18.6 (8.1)	0.27 0.47	Group = 0.01 Session = 0.00 Interaction = 0.24	Group *F*(1, 63) = 18.23, *P* < 0.001 Session *F*(1, 63) = 3.83, *P* < 0.001
Chronic Pain Acceptance Questionnaire Total						
Admission Discharge	64	46.0 (14.8) 59.7 (13.8)	40.4 (16.1) 50.6 (15.2)	0.37 0.63	Group = 0.00 Session = 0.00 Interaction = 0.15	Group *F*(1, 63) = 26.30, *P* < 0.001 Session *F*(1, 63) = 9.34, *P* < 0.001
Sensitivity to Pain Traumatization Scale						
Admission Discharge	45	22.0 (9.5) 18.0 (8.2)	27.0 (11.1) 22.8 (10.5)	0.48 0.51	Group = 0.01 Session = 0.00 Interaction = 0.90	Group *F*(1, 44) = 9.10, *P* = 0.01 Session *F*(1, 44) = 2.72, *P* < 0.001
Pain Stages of Change Questionnaire Pre-contemplation						
Admission Discharge	63	2.9 (0.6) 2.0 (0.6)	3.1 (0.7) 2.5 (0.7)	0.30 0.75	Group = 0.00 Session = 0.00 Interaction = 0.02	Interaction *F*(1, 62) = 7.74, *P* = 0.02
Pain Stages of Change Questionnaire Contemplation						
Admission Discharge	63	3.9 (0.6) 4.1 (0.5)	3.6 (0.7) 3.9 (0.5)	0.41 0.46	Group = 0.00 Session = 0.00 Interaction = 0.76	Group *F*(1,62) = 1.85, *P* < 0.001 Session *F*(1, 62) = 11.27, *P* < 0.001
Pain Stages of Change Questionnaire Action						
Admission Discharge	63	3.0 (0.8) 4.2 (0.4)	3.1 (0.8) 4.0 (0.50)	0.2 0.45	Group = 0.39 Session = 0.00 Interaction = 0.00	Interaction *F*(1, 62) = 1.98, *P* < 0.001
Pain Stages of Change Questionnaire Maintenance						
Admission Discharge	63	3.1 (0.8) 4.3 (0.5)	2.9 (0.8) 3.6 (0.9)	0.16 0.86	Group = 0.61 Session = 0.00 Interaction = 0.06	Session *F*(1, 62) = 1.02, *P* < 0.001
Chronic Pain Coping Inventory Guarding						
Admission Discharge	49	53.0 (7.7) 49.4 (7.3)	55.1 (6.3) 51.3 (7.9)	0.29 0.26	Group = 0.14 Session = 0.00 Interaction = 0.96	Session *F*(1, 48) = 3.03, *P* < 0.001
Chronic Pain Coping Inventory Resting						
Admission Discharge	49	51.0 (7.9) 51.4 (5.8)	56.3 (8.4) 54.6 (9.3)	0.65 0.41	Group = 0.00 Session = 0.51 Interaction = 0.18	Group *F*(1, 48) = 3.69, *P* < 0.001
Chronic Pain Coping Inventory Asking for Assistance						
Admission Discharge	49	48.5 (6.5) 46.7 (7.1)	53.0 (8.1) 51.7 (7.8)	0.61 0.66	Group = 0.00 Session = 0.02 Interaction = 0.78	Group *F*(1, 48) = 0.65, *P* < 0.001 Session *F*(1, 48) = 6.75, *P* = 0.02
Chronic Pain Coping Inventory Exercise/Stretch						
Admission Discharge	49	48.3 (10.3) 60.9 (6.4)	51.8 (9.0) 60.8 (7.6)	0.36 0.02	Group = 0.19 Session = 0.00 Interaction = 0.06	Session *F*(1, 48) = 0.01, *P* < 0.001
Chronic Pain Coping Inventory Relaxation						
Admission Discharge	49	52.9 (8.2) 61.3 (6.2)	53.7 (7.9) 62.6 (7.4)	0.11 0.19	Group = 0.39 Session = 0.00 Interaction = 0.80	Session *F*(1, 48) = 1.69, *P* < 0.001
Chronic Pain Coping Inventory Task Persistence						
Admission Discharge	49	46.9 (8.3) 41.7 (7.1)	41.6 (9.2) 39.9 (8.1)	0.61 0.24	Group = 0.01 Session = 0.00 Interaction = 0.05	Interaction *F*(1, 48) = 0.67, *P* = 0.05
Chronic Pain Coping Inventory Coping Self-Statements						
Admission Discharge	49	47.4 (7.4) 52.8 (7.2)	50.9 (8.5) 54.3 (9.0)	0.44 0.18	Group = 0.07 Session = 0.00 Interaction = 0.18	Session *F*(1, 48) = 1.68, *P* < 0.001
Chronic Pain Coping Inventory Pacing						
Admission Discharge	49	48.2 (7.6) 54.6 (4.9)	52.4 (6.4) 56.2 (6.9)	0.59 0.27	Group = 0.01 Session = 0.00 Interaction = 0.14	Group *F*(1, 48) = 19.11, *P* = 0.01 Session *F*(1, 48) = 4.51, *P* < 0.001
Chronic Pain Coping Inventory Seeking Social Support						
Admission Discharge	49	50.4 (7.0) 50.9 (6.2)	50.9 (7.5) 51.8 (7.4)	0.08 0.13	Group = 0.55 Session = 0.29 Interaction = 0.79	

^a^Number of matched pairs.

Group = Group (veteran/nonveteran) difference; Session = Session (admission/discharge) difference; Interaction = Group by session interaction (combination of group and session differences).

MANOVA results demonstrated main session effects—that is, significant improvements between admission and discharge—for the CES-D, PDI, CPAQ, SPTS, PSOCQ, and CPCI. Main session effects indicate effectiveness of the program for both veterans and nonveterans on average.

Main group effects and group by session interaction effects were also found for the PCS, CAS, PQ, TSK, PSOCQ, and CPCI, providing insight into the differences in pain experiences between veterans and nonveterans. PCS post hoc analysis found a significant session difference for veterans only and a significant group difference at discharge only. Analysis of the CAS yielded a significant session difference only within the veteran group. For the PQ, a significant improvement was found within the veteran group only. TSK analysis yielded a significant difference within the veteran group only by post hoc analysis. Though significant effects were found for all four subscales of the PSOCQ, group by session interactions were only found for Pre-contemplation and Action. For the Pre-contemplation subscale, the interaction was attributed to significant differences within and between groups at discharge only. For the Action subscale, the interaction was attributed to significant differences within both groups. Though main group and/or session effects were found for all CPCI subscales, a group by session interaction was found only for the Task Persistence subscale. Post hoc analysis showed that this interaction was attributed to significant differences within the veteran group only and between groups at admission only.

Table 3 shows *t* test results, which demonstrate that veterans and their case managers perceived greater improvement on average compared to nonveterans on several discharge questionnaires (*P < *0.05).

**Table 3.** Comparison of discharge questionnaire scores between veterans and nonveterans

Discharge questionnaire	*n*^a^	Veteran average (SD)	Nonveteran average (SD)	Cohen’s *d*	*P*
Pain Program Satisfaction Questionnaire	63	37.0 (5.6)	35.5 (5.6)	0.25	0.19
Self-Evaluation Scale	63	3.6 (0.9)	3.2 (1.1)	0.39	0.02*
Patient Evaluation Scale^b^	55	3.7 (0.8)	3.2 (0.9)	0.57	0.00*
Patient Evaluation of Program Benefit					
Physical domain	62	6.3 (2.0)	6.3 (2.3)	0	1.00
Emotional/mental domain	61	6.7 (1.8)	6.6 (2.4)	0.02	0.89
Social domain	54	6.6 (2.1)	6.4 (1.9)	0.09	0.61
Case Manager Evaluation of Program Benefit					
Physical domain	54	6.5 (1.4)	5.8 (2.1)	0.40	0.02*
Emotional/mental domain	52	6.7 (1.6)	5.9 (2.0)	0.43	0.02*
Social domain	54	6.7 (1.8)	6.0 (1.8)	0.39	0.04*

^a^Number of matched pairs.

^b^Case manager’s evaluation of the patient.

*Indicates a significant result.

Comparison of MMPI-2 scores revealed only one significant difference between veterans and nonveterans (see Appendix C). In women, the only MMPI-2 subscale that demonstrated a significant difference was the Self-Presentation (S) validity scale (*P = *0.04). However, both mean values (46.0 for veterans and 43.9 for nonveterans) are below the clinical cutoff of 65; thus, this result is not clinically significant. Additionally, no significant main group effect was found (*P > *0.05), indicating that at admission, veterans and nonveterans are comparable.

## Discussion

The hypothesis that veterans and nonveterans were expected to demonstrate improvements in pain-related domains at discharge was supported by the results of the present study. Significant improvements in depressive symptoms, pain-related disability, pain acceptance, sensitivity to pain traumatization, stages of change, and a number of pain coping domains were found among veterans and nonveterans. Veterans experienced significantly greater improvements than nonveterans in anxiety, pain catastrophizing, recent bothersome symptoms, kinesiophobia, task persistence, and pre-contemplation and action stages of pain.

Among all patients, lower levels of depressive symptoms were observed at discharge from the program. This improvement can be attributed to the integration of activities designed to reduce depression symptoms in the 4-week intensive MGD program. On average, all patients also endorsed lower pain-related interference in family and home responsibilities, recreation, social activity, occupation, sexual behavior, self-care, and life support activity at discharge. These findings align with previous research, because depressive symptoms and disability have been shown to decrease after attending similar pain management programs.^12,40^

All patients improved on average on all four pain stages of change, essential in pain management, because changes in patients’ attitudes in adopting a pain self-management approach can predict long-term function.^41^ For the pre-contemplation and action stages, veterans demonstrated significantly greater improvements than nonveterans. Reductions in pre-contemplation are predictive of lower depressive symptoms, and increases in action are predictive of lower pain severity.^41^

Patient adjustment can be further predicted by patients’ scores on the coping strategies of guarding, asking for assistance, relaxation, and task persistence (CPCI).^42^ Improvements were observed for all patients on all of these scales except for task persistence. For task persistence, only veterans changed at discharge. Their higher scores at admission may be due to their need to complete tasks irrespective of pain given their extensive military disciplinary training. Their reduction in scores at discharge reflects a positive and adaptive change for veterans.

Both veterans and nonveterans also improved significantly on their sensitivity to pain traumatization as indicated by the differences in SPTS scores between admission and discharge. Nonveterans had higher SPTS scores at admission and discharge, aligning with the fact that there were significantly more PTSD diagnoses among veterans in this study. Moreover, this finding is consistent with similar differences in anxiety symptoms, because the SPTS focuses on anxiety-related cognitive, emotional, and behavioral reactions to pain.^12^ Anxiety levels among veterans decreased on average at discharge from the program, whereas nonveterans made a smaller, nonsignificant improvement. A potential source of anxiety for nonveterans could be anticipating returning to work, considering that their continued chronic pain could be a source of anxiety, because only seven veterans and 23 nonveterans were employed at admission. Moreover, nonveterans may also be experiencing their unresolved litigation cases as sources of stress and anxiety.

The finding of improvement in pain catastrophizing in all patients supports the literature, because behavioral interventions have been shown to decrease negative psychological states, including depression and pain catastrophizing.^40^ Significantly greater improvements in pain catastrophizing among veterans suggest that being a veteran may influence perspectives on pain, explaining the difference between groups. Small to moderate benefits for catastrophic thinking in addition to depression, anxiety, and disability have been reported in the literature.^41^ A potential explanation for the significantly greater improvements made by veterans at discharge could be a result of greater pain chronicity. Because veterans have, on average, experienced chronic pain for a significantly greater amount of time compared to nonveterans, they may have developed strategies to stabilize negative mindsets. This hypothesis is also supported by our pain acceptance findings. Even though both veterans and nonveterans showed an increase in acceptance of their chronic pain condition, a greater increase in pain acceptance was found among veterans. The difference in pain chronicity between groups may explain the demographic difference that veterans are higher users of primary care in comparison to nonveterans, as displayed in Table 1. Because veterans have had pain for longer and their pain is often more severe and complex, with comorbidities such as PTSD, a greater number of medical visits is expected.^43^

As a result of the teachings and practices integrated within the 4-week MGD program such as fitness education and daily fitness sessions, the finding that all participants reported lower levels of kinesiophobia at discharge is expected.^44^ Even though improvement in kinesiophobia has also been demonstrated in previous research, the present study found a greater difference in kinesiophobia scores among veterans.^12^ This difference may be explained by demographic differences in employment status and the risk of injury at work, because a higher proportion of nonveterans than veterans were employed. Lower levels of fear of re-injury among veterans may be because the majority are retired and do not expect to return to work.

Aligning with the expectation that patients would have an improved experience with their pain condition as a result of the program, both veterans and nonveterans endorsed lower levels of recent bothersome symptoms at discharge. However, a significant difference in scores between admission and discharge was only found in the veteran group, indicating that, on average, the program was especially effective for veterans. These results add to the literature, because Jiwani and Hapidou’s study found no significant improvements in bothersome symptoms for veteran and nonveteran groups.^12^

Program evaluation scores were found to be highly correlated between patients and their case managers (see Appendix D), providing evidence that these discharge questionnaires are valid evaluations of improvement at the end of the program. Both veterans and nonveterans improved in pain-related psychological measures, and self-reported evaluations also attested to these improvements. No differences were found between how veterans and nonveterans evaluated their own improvement in physical, emotional/mental, and social domains. However, case managers evaluated veterans as showing greater improvement than nonveterans on these domains. On the other hand, there was no difference in patient satisfaction between veterans and nonveterans, ultimately supporting that, irrespective of referral source, the 4-week MGD program is effective for all participants in this sample.

A strength of this study is that a variety of psychometric measures were utilized to assess pain on multiple dimensions, prospectively providing clinicians with a comprehensive evaluation of patients’ progress through the 4-week program. By comparing veteran pain outcomes to those of their nonveteran counterparts, this study contributes to the literature because results align with previous findings and provide more insight into pain management among veterans.^12,40^

Limitations of the present study include unknown confounders due to the reliance on retrospective sources of data. However, it was not feasible to conduct this study as a randomized controlled trial because the independent variable of group membership (veteran or nonveteran) cannot be randomized. Additionally, self-reported data are subject to misreporting, resulting in bias. Thus, further research should include objective measures such as functional magnetic resonance imaging or physical therapy outcomes.

Even though the psychometric measures utilized in this study are all valid and reliable, for the CES-D measure, an amended analysis could have been conducted to exclude several items that have demonstrated psychometric difficulties in previous studies, as recommended by Carleton et al.^45^ Additionally, the sample size of this study was limited to 136 patients due to the number of veterans who completed the program. The small sample size relative to the high number of variables examined may have limited the results of the study by introducing potential selection bias, impacting the generalizability of findings. Thus, this research would benefit from a replication study with a larger sample size. A follow-up study is currently underway on the 4-week MGD program to determine the extent to which treatment effects persist over time.

## Conclusion

The current study contributes to the literature by providing evidence of the effectiveness of interdisciplinary pain management programs in addressing chronic pain and related comorbidity in veterans and nonveterans. Additionally, the unique facets of pain experience among veterans were identified, aiding clinicians in offering better informed approaches to pain management for this population, thus improving the lives of veterans and their families.

## Appendix A.

**Table A1.** Post hoc multiple comparisons analysis

Measure	Scheffe tests	Difference	Comparison values (absolute)
PCS	8.26	Veterans: Session	14.39*
		Nonveterans: Session	7.64
		Admission: Group	2.69
		Discharge: Group	9.44*
CAS	9.84	Veterans: Session	12.51*
		Nonveterans: Session	4.67
		Admission: Group	0.35
		Discharge: Group	7.96
PQ	2.62	Veterans: Session	3.59*
		Nonveterans: Session	1.68
		Admission: Group	0.48
		Discharge: Group	1.43
TSK	8.49	Veterans: Session	10.34*
		Nonveterans: Session	3.41
		Admission: Group	1.27
		Discharge: Group	5.66
PSOCQ Pre-contemplation	0.37	Veterans: Session	0.86*
		Nonveterans: Session	0.55*
		Admission: Group	0.20
		Discharge: Group	0.51*
PSOCQ Action	0.55	Veterans: Session	1.33*
		Nonveterans: Session	0.88*
		Admission: Group	0.15
		Discharge: Group	0.30
CPCI Task Persistence	4.249	Veterans: Session	5.14*
		Nonveterans: Session	1.69
		Admission: Group	5.31*
		Discharge: Group	1.86

* Indicates a significant difference.

PCS = Pain Catastrophizing Scale; CAS = Clinical Anxiety Scale; PQ = patient questionnaire; TSK = Tampa Scale of Kinesiophobia; PSOCQ = Pain Stages of Change Questionnaire; CPCI = Chronic Pain Coping Inventory.

## Appendix B.

**Table B1.** Chronicity correlations

	PCSD	CPAQAD	CPAQPA	CPAQTD	PSOCQPD	PSOCQCD	SPTSD
Chronicity	−0.25**	0.26**	0.18*	0.20*	−0.23*	0.22*	−0.24*

* Correlation is significant at the 0.05 level.

** Correlation is significant at the 0.01 level.

PCSD = Pain Catastrophizing Scale at discharge; CPAQAD = Chronic Pain Acceptance Questionnaire Activities Engagement at discharge; CPAQPA = Chronic Pain Acceptance Questionnaire Pain Willingness at admission; CPAQTD = Chronic Pain Acceptance Questionnaire Total at discharge; PSOCQPD = Pain Stages of Change Questionnaire Pre-contemplation at discharge; PSCOCQCD = Pain Stages of Change Questionnaire Contemplation at discharge; SPTSD = Sensitivity to Pain Traumatization Scale at discharge.

## Appendix C.

**Table C1.** MMPI-2 subscale scores

Scale	*n*	Veteran average (SD)	Nonveteran average (SD)	*P*
VRIN	45 7	Men = 54.00 (11.88) Women = 52.29 (6.05)	Men = 52.13 (9.29) Women = 57.43 (10.94)	0.64 0.14
F	45 7	Men = 65.84 (15.63) Women = 69.71 (13.43)	Men = 67.78 (15.29) Women = 67.29 (14.07)	0.53 0.86
Fb	45 7	Men = 62.69 (20.38) Women = 64.71 (19.92)	Men = 64.93 (20.61) Women = 65.14 (20.65)	0.15 0.52
Fp	45 7	Men = 50.29 (12.14) Women = 61.57 (12.09)	Men = 54.31 (11.83) Women = 53.57 (10.18)	0.33 0.81
FBS	45 7	Men = 77.36 (16.88) Women = 80.86 (12.60)	Men = 82.42 (14.23) Women = 82.00 (8.35)	0.50 0.29
L	45 7	Men = 53.76 (11.25) Women = 50.00 (8.35)	Men = 59.58 (9.87) Women = 51.43 (8.98)	0.87 0.98
K	45 7	Men = 46.62 (10.56) Women = 46.14 (12.08)	Men = 46.00 (8.51) Women = 44.57 (10.63)	0.87 0.90
S	45 7	Men = 44.93 (9.10) Women = 46.00 (11.71)	Men = 44.76 (9.96) Women = 43.86 (10.59)	0.84 0.04*
Hs	45 7	Men = 80.04 (14.17) Women = 81.86 (11.07)	Men = 84.47 (10.69) Women = 81.86 (8.29)	0.28 0.51
D	45 7	Men = 78.58 (14.39) Women = 83.29 (17.85)	Men = 84.89 (13.32) Women = 86.00 (10.25)	0.97 0.09
Hy	45 7	Men = 79.58 (14.45) Women = 80.86 (14.59)	Men = 85.60 (16.04) Women = 87.14 (15.05)	0.36 0.35
Pd	45 7	Men = 64.71 (12.83) Women = 67.86 (6.49)	Men = 63.64 (11.21) Women = 67.86 (6.49)	0.25 0.37
Mf	45 7	Men = 48.11 (7.66) Women = 62.00 (11.82)	Men = 48.22 (9.72) Women = 50.00 (10.65)	0.33 0.31
Pa	45 7	Men = 65.82 (17.05) Women = 67.71 (14.06)	Men = 67.64 (15.55) Women = 64.57 (11.18)	0.46 0.79
Pt	45 7	Men = 74.76 (15.87) Women = 74.57 (11.28)	Men = 76.78 (14.54) Women = 73.57 (5.88)	0.96 0.26
Sc	45 7	Men = 76.24 (16.88) Women = 78.86 (9.34)	Men = 74.87 (16.67) Women = 76.00 (9.50)	0.94 0.94
Ma	45 7	Men = 56.16 (13.96) Women = 57.71 (15.77)	Men = 53.02 (9.37) Women = 50.43 (8.56)	0.13 0.49
Si	45 7	Men = 57.36 (12.91) Women = 60.57 (15.09)	Men = 60.87 (10.39) Women = 59.71 (8.90)	0.59 0.99

* Indicates significant result (*P < *0.05).

MMPI-2 = Minnesota Multiphasic Personality Inventory-2; VRIN = Variable Response Inconsistency; F = Infrequency; Fb = Back F; Fp = Psychopathology; FBS = Symptom Validity; L = Lie; K = Correction; S = Superlative Self-Presentation; Hs = Hypochondriasis; D = Depression; Hy = Hysteria; Pd = Psychopathic Deviate; Mf = Masculinity/Femininity; Pa = Paranoia; Pt = Psychasthenia; Sc = Schizophrenia; Ma = Hypomania; Si = Social Introversion.

## Appendix D.

**Table D1.** Benefit measure correlations

	PSES	CSES	PPSQ	PPB	PEB	PSB	CPB	CEB	CSB
PSES	1								
CSES	0.515	1							
PPSQ	0.470	0.369	1						
PPB	0.421	0.361	0.420	1					
PEB	0.461	0.309	0.416	0.628	1				
PSB	0.458	0.330	0.430	0.563	0.610	1			
CPB	0.440	0.752	0.341	0.410	0.273	0.238	1		
CEB	0.480	0.763	0.357	0.396	0.382	0.305	0.740	1	
CSB	0.422	0.650	0.380	0.345	0.378	0.427	0.659	0.768	1

PSES = patient Self-Evaluation Scale; CSES = case manager Self-Evaluation Scale; PPSQ = Pain Program Satisfaction Questionnaire; PPB = patient physical benefit; PEB = patient emotional benefit; PSB = patient social benefit; CPB = case manager physical benefit; CEB = case manager emotional benefit; CSB = case manager social benefit.
